# A comparative study of oxidative stress and interrelationship of important antioxidants in haloperidol and olanzapine treated patients suffering from schizophrenia

**DOI:** 10.4103/0019-5545.43627

**Published:** 2008

**Authors:** Om Prakash Singh, Indranil Chakraborty, Anindya Dasgupta, Subinay Datta

**Affiliations:** Department of Psychiatry, Burdwan Medical College, Burdwan - 713 104, West Bengal, India; 1 Department of Biochemistry, Burdwan Medical College, Burdwan - 713 104, West Bengal, India

**Keywords:** Antipsychotics, oxidative stress, schizophrenia

## Abstract

**Context:**

Oxidative stress induced lipid peroxidation has been a significant contributing factor for schizophrenia. Older antipsychotics, like haloperidol , were found to increase lipid peroxidation, whereas, the newer atypical antipsychotics, like olanzapine, did not generate free radicals as metabolic end products.

**Aims:**

The interrelationship of the antioxidant vitamins and antioxidant enzymes, and their overall effect on regulation of oxidative stress induced by haloperidol as compared to olanzapine were analyzed in present study.

**Setting and Design:**

It was an open randomized cross sectional study that consisted of two groups of fifty schizophrenic patients treated by haloperidol and olanzapine, respectively for at least six months.

**Materials and Methods:**

Serum thiobarbituric acid reacting substances (TBARS) was selected as marker of lipid peroxidation, whereas, serum tocopherols, plasma ascorbate and plasma superoxide dismutase (SOD) activity, were selected to assess the antioxidant vitamin and antioxidant enzyme status, respectively. All measurements were done by standard photometric methods.

**Statistical Analysis Used:**

Statistical analysis was performed to find out the significance for the differences of means between two groups. Bivariate and partial correlation coefficients for assessing the interrelationship between different parameters were done by using statistical package for social sciences (SPSS) software.

**Results:**

Results showed significantly higher serum TBARS and lower antioxidant values in the haloperidol treated patients. Significant positive correlations among the individual antioxidant parameters and significant negative correlation between all of the antioxidant parameters and serum TBARS were found only in haloperidol treated patients. Plasma SOD activity correlated to plasma ascorbate in both groups. Partial correlation results revealed that the serum tocopherol decreased linearly with an increase in serum TBARS significantly in olanzapine treated patients when effect of plasma ascorbate was controlled.

**Conclusions:**

Haloperidol caused more oxidative stress along with a significant reduction of important antioxidant parameters. Plasma ascorbate was found to be the chief antioxidant on which the activity of both plasma SOD and alpha tocopherol were dependent under oxidative stressful conditions.

## INTRODUCTION

Schizophrenia afflicts approximately 1% of world population and is supposed to be a multifactorial disorder. Oxidative stress due to an impaired redox balance has been supposed to be one of the major causes for schizophrenia.[1,2] Increased generation of free radicals occurs due to unbalanced oxidative stress. Shifting of the redox balance towards oxidative stress may occur due to excess generation of free radicals, decreased antioxidant activities, or a combination of both. Brain cell membranes constitute greater than 66% phospholipids by mass versus 33% in peripheral tissues.[3] The R_2_ positions in all of these phospholipids are occupied exclusively by essential polyunsaturated fatty acids (EPUFA), which are susceptible to breakdown by increased oxidative stress, thus generating lipid peroxidation products. Moreover, during neurotransmission also the catecholamines generate reactive oxygen species (ROS), which play a large role in n-methyl D-aspartate (NMDA) and non-NMDA mediated glutamate excitotoxicity.[4] Antipsychotics used to treat schizophrenia have been found to have either pro-oxidant or antioxidant effects on cell membrane lipids, based on the differences in their chemical properties.[5–8] The older antipsychotics which are used in treatment in schizophrenia, are supposed to cause increased generation of free radical within the brain.[9–13] Haloperidol, that blocks the D_2_ receptors in brain has been the preferred drug for acute schizophrenia for a long period, but it has been found to be converted to a free radical in the brain, and that free radical is found to cause neural damage.[14] Still there exists a lacuna in the knowledge of how the significant improvement in the symptoms of schizophrenia in the haloperidol treated patients persists even after an increased free radical production by the drug itself. Most probably, the free radical induced damages are reflected by increased side effects like tardive dyskinesia, associated with the treatment with conventional antipsychotics like haloperidol . On the other hand, the newer atypical antipsychotics are not found to cause generation of free radicals as their metabolic end products, and so are supposed to be better treatment alternatives nowadays. Olanzapine is such a new atypical antipsychotic blocking multiple monoaminergic (D_2_, 5HT_2_, α_1_, α_2_) as well as muscarinic and H_1_ receptors. Along with olanzapine, several atypical antipsychotics have been found to increase membrane phospholipids and EPUFAs particularly arachidonic acid (AA) and docosahexanoic acid (DHA).[15–17] These changes correlate with improved psychopathology in schizophrenia. Although the exact mechanisms of their action are still unclear, it has been suggested that atypical antipsychotics might inhibit the phospholipases, increase the antioxidant defense enzymes and do not cause lipid peroxidation similar to haloperidol.[18] The roles of some antioxidant vitamins as well as antioxidant enzymes, as additive factors in the alleviation of oxidative stress have been encouraging. Studies have suggested beneficial roles of ascorbate[19] and alpha tocopherol[20] in the management of oxidative stress in the schizophrenia along with the atypical antipsychotics. Keeping these facts in mind, the present study was undertaken in the Psychiatry and Biochemistry department of Burdwan Medical College and Hospital, West Bengal, to analyze the interrelationship of the antioxidant vitamins and antioxidant enzymes, and their overall effect on regulation of oxidative stress induced by the older antipsychotic drugs like haloperidol as compared to the newer antipsychotics like olanzapine.

## MATERIALS AND METHODS

The present hospital based and open randomized study was a cross sectional type that was undertaken in the Department of Psychiatry and Department of Biochemistry of Burdwan Medical College and Hospital, a tertiary care hospital in West Bengal. Being the only tertiary care level hospital in the whole district of Burdwan, with a fully functional Psychiatric out patient department (OPD) and indoor ward at government level, the patients attending this hospital from distant places comprised of a suitable target population for the present study.

The study was conducted during the period from March 2005 to March 2006. The cases were selected from the schizophrenic patients attending the outdoor of Psychiatry Department. Diagnosis of schizophrenia was made by using the ICD-10 criteria.[21] The target patients were selected in two groups. In Group I those schizophrenic patents were selected who were being treated by the drug haloperidol for at least 6 weeks and in the Group II those were selected who were being treated by the newer antipsychotic drug, olanzapine for the same duration. Dosage of both drugs was titrated during the course of treatment to obtain maximum clinical benefit. For haloperidol , the required dosage was found to be 5-15 mg per day with an average of 10 mg per day. These patients did not receive any other drug during this period except trihexyphenidyl given occasionally in required dosage (4-6 mg/day) to combat the extrapyramidal side effects. None of them were suffering from any other metabolic or chronic disorder at the time of presentation. Fifty patients were selected by a simple random method from a total number of five hundred patients treated by haloperidol and fulfilling the above criteria during the period of one year. This comprised Group I. Twenty six of them were males and the rest were females. For olanzapine, this dose range was 5-20 mg per day with an average of 12.5 mg per day. These patients also did not receive any other drug during this period except trihexyphenidyl given occasionally in required dosage (4-6 mg/day) to combat the occasional extrapyramidal side effects. None of them were suffering from any other metabolic or chronic disorder. Fifty patients were selected by a simple random method from a total number of two hundred and sixty patients being treated by olanzapine and fulfilling the above criteria during the period of one year and thus comprised the Group II. Twenty five of them were males and the rest were females. Cases from both categories were selected from similar socioeconomic status and ethnicity with similar nutritional and dietary habits. None of them were treated with any vitamin or nutritional supplement during the period of antipsychotic treatment. The mean age for the patients treated by haloperidol was 26.92 years with an SD of 3.39 and that for the patients treated by olanzapine was 25.46 years with an SD of 3.57. The age difference between these two groups was found to be statistically insignificant (*P* = 0.511) and so, the groups were age matched. Both categories were informed about the risks and benefits of the study and written consents were obtained from their attending relatives before drawing blood from the patients. The study was approved by a properly constituted institutional ethical committee. The overall study was carried out in accordance with the Helsinki Declaration 1975.

Serum thiobarbituric acid reacting substances (TBARS) was selected as a marker of lipid peroxidation. Estimations of serum tocopherols and plasma ascorbate were done to assess the antioxidant vitamin status. On the other hand, estimation of plasma superoxide dismutase (SOD) activity was done to assess the antioxidant enzyme status.

Estimation of serum TBARS (Thiobarbituric acid test):- TBARS was measured in serum by its reaction with thiobarbituric acid (TBA).[22] Briefly, 0.5 ml of freshly prepared serum was added to 2.5 ml of 20% trichloroacetic acid, 2.5 ml of 0.05M sulphuric acid was added to it after 10 min, and 3.5 ml of TBA reagent was added. Heating in boiling water bath for 30 min carried out the coupling of lipid peroxide with TBA. After cooling in water the chromogen was extracted by adding 4.0 ml of *n*-butanol. Its absorbance was determined at 532 nm wavelength by spectrophotometer (spectronic-21). Level of TBARS was calculated from standard curve prepared from 5 nmol/ml, 7.5 nmol/ml, 10nmol/ml, 12.5 nmol/ml, 15 nmol/ml, 17.5 nmol/ml and 20 nmol/ml of 1,1,3,3 tetraethoxypropane (from Fluka, Germany). Serum TBARS values were expressed in nmol/ml.

Serum tocopherols estimation:- Serum tocopherols were measured by their reduction of ferric to ferrous ions which then formed a red color complex with α,α '-dipiridyl.[23] Briefly, beta carotene being lipid soluble were first extracted into xylene and the absorbance was read at 460 nm. 1.5 ml of serum, standard (Standard solution of DL-α -tocopherol from Cigma, USA, 10 mg/L in ethanol) was taken and 1.5 ml of ethanol (aldehyde free absolute alcohol) was added to the test and 1.5 ml of water to the standard tube. 1.5 ml of xylene was added in all, the contents and centrifuged, and 1 ml of xylene layer from each was transferred into clean, stoppered tube excluding any protein or ethanol. 1 ml of dipiridyl reagent (α,α′- dipiridyl, 1.2 gm/L in 1-propanol) was added to each, 1.5 ml of the mixture were pipetted into colorimeter cuvettes and absorbance read at 460 nm against blank. 0.33 ml of ferric chloride solution (0.12 percent FeCl_3_.6H_2_O in ethanol) was added and absorbances were taken at 520 nm against blank. Correction for the beta carotene was made by subtracting the absorbance at 460 nm from that at 520 nm, and concentration of tocopherol was calculated. Results were expressed in mg/L.

Determination of plasma ascorbic acid (Photometric method):- Plasma ascorbate was oxidized by Cu^2+^ to form dehydroascorbic acid, which reacted with 2, 4 dinitrophenylhydrazine to form a red bis-hydrazone, which was measured at 520 nm.[24] The analysis was done from the heparinized plasma immediately. Briefly, 0.5 ml of heparinized plasma was added to 2 ml of freshly prepared 20% trichloroacetatic acid (TCA), mixed well in a vortex mixture, and centrifuged for 10 min at 2500*g*. 1.2 ml of supernatant was taken in a screw-capped test tube, 0.4 ml of diphenylhydrazine-thiourea-copper sulphate (DTCS) reagent was added and the tubes were incubated at 37°C in a waterbath for 3 h. After removal from the waterbath and chilling in ice bath for 10 min, 2 ml of 12 M/L cold H_2_SO_4_ was added slowly, the contents were mixed and the absorbances were recorded at 520 nm. Final concentrations of the samples were calculated by calibrating them against the standard curve of ascorbate obtained by plotting the absorbances against the respective concentrations of 0.1 mg/dl, 0.4 mg/dl, 0.8 mg/dl, 1.2 mg/dl, 2 mg/dl, 3 mg/dl, and 4 mg/dl of L ascorbate solutions in water.

Estimation of plasma SOD activity:- Estimation of plasma SOD was done by the method of Kakkar *et al.*[25] 1.35 ml of double distilled water, 50 µl of plasma, 1.2 ml of sodium pyrophosphate buffer (pH 8.3), 0.1 ml of phenazine methosulphate (PMS) and 0.3 ml of nitroblue tetrazolium (NBT) were mixed. 0.2 ml of NADH solution was added to it to initiate the reaction. After incubation at 39°C for 90 s the reaction was terminated by adding 1 ml of glacial acetic acid. 4 ml of *n*-butanol was added and the mixture was centrifuged at 4000 rpm for 10 min and the absorbance of the upper butanol layer recorded at 560 nm. For the comparison, corresponding blank was prepared in the same way except addition of the plasma. One unit of SOD was defined as that amount of enzyme that inhibits the rate of reactions by 50% under specified conditions.

The study was designed to find the interrelationship of the different antioxidants and their effect on the oxidative stress among the schizophrenic patients treated with haloperidol and olanzapine of this district from the same area. Hence, the data obtained were first analyzed for differences of means to evaluate the degree of differences in the oxidative stress and antioxidant parameters between two groups; and then for analyses of bivariate correlation coefficients and partial correlation coefficients to assess the interrelationship between different parameters. All statistical analyses were done using statistical package for social sciences (SPSS) software.

## RESULTS

From the Table 1, it is evident that serum TBARS was significantly higher in the haloperidol treated patients, whereas in the same group, all of the antioxidant parameters were found to be significantly lower. Table 2 shows correlation studies where the linear relationship between the individual antioxidant parameters as well as the relationship between each of them with the lipid peroxidation product has been shown. Significant positive correlation was observed among the individual antioxidant parameters in both groups, except that between the serum alpha tocopherol and plasma SOD in the haloperidol treated patients. In contrast, a significant negative correlation was found between the antioxidant parameters and the lipid peroxidation product, i.e. serum TBARS in the haloperidol treated patients. But, correlation between the serum alpha tocopherol and plasma ascorbate with serum TBARS was not significant in the olanzapine treated group. Partial correlation studies in the Table 3 showed the strength of linear relationship between the important parameters after controlling or withdrawing the effect of the other ones. For example, the linear relationship between serum TBARS and serum tocopherol (*X*_13_), which was insignificant in bivariate correlation study for the olanzapine treated group as found in Table 2, was found to be significantly negative after controlling or withdrawing the effect of plasma ascorbate (*X*_13. 2_) in the Table 3. In contrary, the linear relationship between serum tocopherol and plasma SOD (*X*_34_), which was insignificant for the haloperidol treated group in bivariate correlation study [Table 2], became significantly positive when the effect of plasma ascorbate was withdrawn or controlled for (*X*_34. 2_) in the Table 3.

**Table 1:** Independent *t*-test for differences between means of the variables from haloperidol and olanzapine treated patients of schizophrenia

Variables	Mean + SD for haloperidol	Mean + SD for olanzapine	*t* value	Significance (2-tailed)
Plasma ascorbate (mg/dl)	0.55 + 0.09	0.82 + 0.05	−17.62	*P* < 0.001
Serum TBARS (nmol/ml)	6.09 + 0.41	3.79 + 0.63	21.5	*P* < 0.001
Plasma SOD (IU/ml)	5.69 + 1.2	7.60 + 1.33	−7.4	*P* < 0.001
Serum tocopherol (mg/l)	8.65 + 0.71	10.56 + 0.43	−16.2	*P* < 0.001

Statistical analysis done by SPSS software.

**Table 2:** Bivariate correlation analyses for the variables from haloperidol and olanzapine treated patients of schizophrenia. Serum TBARS = *X*_1_, plasma ascorbate = *X*_2_, serum tocopherol = *X*_3_, plasma SOD = *X*_4_.

Variables	Correlation coefficient (r) for haloperidol treated patients	Correlation coefficients for olanzapine treated patients
*X*_1.2_	*r* = −0.955**	*r* = −.080***
*X*_1.3_	*r* = −0.545**	*r* = −0.020***
*X*_1.4_	*r* = −0.597**	*r* = −0.284*
*X*_2.3_	*r* = 0.420**	*r* = 0.950**
*X*_2.4_	*r* = 0.606**	*r* = 0.388**
*X*_3.4_	*r* = 0.168***	*r* = 0.322*

*r* = Correlation coefficients.

* Correlation is significant at 0.05 level (2-tailed).

** Correlation is significant at 0.01 level (2-tailed).

*** Correlation is insignificant (*P*> 0.05, 2-tailed). Statistical analysis done by SPSS software.

**Table 3:** Partial correlation coefficients (*r*) describing the linear relationship between two variables while controlling for the effects of one or more additional variables. Serum TBARS = *X*_1_, plasma ascorbate = *X*_2_, serum tocopherol = *X*_3_, plasma SOD = *X*_4_.

Variables	Partial correlation coefficients for haloperidol treated patients	Partial correlation coefficients for olanzepine treated patients
*X*_13. 2_	*r* = −0.534**	*r* = −0.307*
*X*_12. 3_	*r* = −0.953**	*r* = 0.316*
*X*_34. 2_	*r* = 0.480**	*r* = −0.173***

*r* = Partial correlation coefficient.

* Correlation is significant at 0.05 level (2-tailed)

** Correlation is significant at 0.001 level (2-tailed)

*** Correlation is statistically insignificant (*P* > 0.05, 2-tailed); Statistical analysis done by SPSS software.

## DISCUSSION

Analyses of data from the present study suggested that there was a significant increase in lipid peroxidation in the schizophrenic patients treated with the older antipsychotic drug haloperidol , in comparison to those treated with the newer antipsychotic drug olanzapine. Increased lipid peroxidation, in turn, was possibly due to a deranged redox balance as evident from significantly reduced levels of all antioxidant parameters in the group treated by haloperidol [Table 1]. This significant reduction in their levels, most probably, was due to a significant increase in oxidative stress in the patients treated with haloperidol as stated earlier in some other studies as well.[14] As both groups were selected from the same area with comparable economic, ethnic and social status with grossly same food habits, and none of them suffered from any other chronic or metabolic disease, the increase in oxidative stress in the present study population treated with haloperidol could supposed to be due to the drug itself. This was supported by the data in Table 2 where the serum TBARS was significantly negatively correlated to all antioxidants in the patients treated with haloperidol. But in the group treated with olanzapine, serum TBARS was not found to be correlated significantly to the plasma ascorbate and serum tocopherol that signified the absence of increased oxidative stress induced consumption of these antioxidants in these patients. But in the partial correlation study [Table 3] the linear relationship between the alpha tocopherol and TBARS in serum became significantly negative in the olanzapine treated patients also when the effect of plasma ascorbate was controlled or withdrawn (*X*_13. 2_). These findings suggested that for maintenance of the normal level of alpha tocopherol a normal level of plasma ascorbate was also necessary. These findings correlate well with the fact that in lipid peroxidation, when alpha tocopherol attenuates the process by breaking the chain of free radical propagation, ascorbic acid helps in the regeneration of the alpha tocopherol activity.[26] Thus, it was clearly indicated that there was an increased oxidative stress induced lipid peroxidation in the patients treated with haloperidol that resulted in increased consumption of the antioxidant vitamins leading to their significant reduction. Moreover, due to a prevailing decreased ascorbate level in them, oxidatively modified, inactive alpha tocopherol could not be regenerated into its active form. This further exaggerated the oxidative stress induced lipid peroxidation in the brain cell membranes.

Plasma SOD activity also showed a significant negative correlation to the serum TBARS in both groups but, the negative correlation and its significance was greater in the group treated with haloperidol [Table 2]. This suggested a reduction in the level of this antioxidant enzyme against lipid peroxidation, which was more in haloperidol treated group due to a significant increase in the oxidative stress. The observations of the present study correlated well with some other studies where Cu^2+^-Zn^2+^ SOD, a chief regulator of oxidative stress, was found to be compromised with an increased oxidative stress in the schizophrenic patients.[27] As cytoplasmic SOD, extracellular SOD is also a Cu^+^Zn^+^ dependent enzyme and its activity has been found to correlate well with the cytoplasmic SOD activity in RBC in some studies,[28] it reflected the status of intracellular SOD activity as well in our present study. Moreover, a significantly positive correlation of plasma SOD activity to the plasma ascorbate in both groups [Table 2] indicated a dependence of the activity of this antioxidant enzyme on ascorbate level under conditions of both increased and decreased oxidative stress. But, the correlation of the plasma SOD activity to serum tocopherol was not significant for the patients treated with haloperidol although this was significantly positive in the patients treated with olanzapine. It became significantly positive for the haloperidol treated group only when the effect of the plasma ascorbate was controlled or withdrawn. Thus, it strongly suggested that when both antioxidant vitamins were present, in statistical consideration plasma SOD level was chiefly dependent on plasma ascorbate level under the oxidative stressful condition produced by haloperidol treatment. It showed significant dependence on alpha tocopherol only when the effect of plasma ascorbate was controlled or withdrawn (X_34. 2_ in Table 3) in the same group. Thus, data analyses suggested that plasma SOD activity became dependent primarily on the plasma ascorbate level in the schizophrenic patients in the present study. The free radical scavenging function of ascorbate most probably protects the SOD enzyme integrity and activity against the free radical induced damages. Ascorbate has been found to be the principal antioxidant in the brain serving to scavenge the ROS generated by glutamate receptor in earlier studies as well.[29] Ascorbate was also found as an important antioxidant that prevented dopamine against oxidation by RNS derived from NO.[10] The final observations of the present study indicate that ascorbate possesses the major antioxidant role in schizophrenic patients also.

In conclusion, it can be opined that olanzapine, the newer antipsychotic drug, has a significantly lower chance of producing free radical induced damage during the therapy of schizophrenia than the conventional drug haloperidol. On the other hand, the antioxidant vitamins and plasma SOD, particularly the plasma ascorbate significantly contribute in reducing the oxidative stress produced by the drug haloperidol in the schizophrenic patients. Some previous, as well as, recent studies have pointed out protective roles of the antioxidant enzyme like superoxide dismutase (SOD) activity[27] and the antioxidant vitamins, namely ascorbate and alpha tocopherol under the oxidative stressful conditions found in schizophrenia.[19,20] Hence, from the findings of our present study it can be suggested that administration of ascorbic acid and alpha tocopherol, particularly ascorbic acid might be helpful in reducing the oxidative stress induced damages in those schizophrenic patients who have to be treated by haloperidol for optimum benefit.
